# Oxidative stability and quality characteristics of whey protein coated rohu (*Labeo rohita*) fillets

**DOI:** 10.1186/s12944-015-0060-z

**Published:** 2015-06-23

**Authors:** Muhammad Issa Khan, Muhammad Nawaz Adrees, Muhammad Sajid Arshad, Faqir Muhammad Anjum, Cheorun Jo, Aysha Sameen

**Affiliations:** National Institute of Food Science and Technology, University of Agriculture, Faisalabad, Pakistan; Department of Agricultural Biotechnology, Center for Food and Bioconvergence, Research Institute of Agricultural and Life Science, Seoul National University, Seoul, 151-921 Republic of Korea; Institute of Home and Food Sciences, Government College University, Faisalabad, Pakistan

**Keywords:** Whey protein coating, Fish fillets, TBARS, TVBN, Oxidative stability, Quality

## Abstract

**Background:**

Edible coatings have beneficial effect on quality of fish and act as barrier against moisture transfer and uptake of oxygen. Edible coating made up of biodegradable materials is helpful to control the quality deterioration and enhance the shelf life.

**Methods:**

The present study was designed to elucidate the effects of whey based protein using two plasticizers i.e. sorbitol and glycerol on oxidative stability and quality characteristics of Rohu (*Labeo rohita)*. Coating solutions were prepared by incorporating whey (8 % protein; w/ w) in distilled water followed addition of sorbitol and glycerol. Dipping method was used to apply coating on fish fillets. The coated fillets were subjected to quality characterictics, pH, color, TBARS, peroxide value, volatile basic nitrogen (TVBN) and sensory evaluation during 40 days of storage.

**Results:**

The results showed significant impact on different quality attributes of fish fillets. Highest (TVBN) and TBARS were observed in control samples (T_0_) (12.60 ± 0.25, mg/100 g, 0.820 ± 0.02 mg MDA/kg) while lowest in T_3_ coated samples (8.81 ± 0.18 mg/100 g., 0.352 ± 0.01 mg MDA/kg of meat). Moreover, sensorial findings did not showed adverse effects and T_3_ coated samples were ranked higher by consumers.

**Conclusion:**

In conclusion, coating fish with Whey: Glycerol: Sorbitol (1:1:1) in current investigation enhances the storage life and quality of fish fillets.

## Introduction

Fish provide valuable essential health promoting fatty acids, amino acids, lipid soluble vitamins and micronutrients [1]. Fish is essential part of human diet in many regions of the world and for some countries major source of income [2]. Rohu (*Labeo rohita*) is a fresh water fish of the carp family *Cyprinidae* and ubiquitously present throughout South and South East Asia in the weedy, slow flowing or standing waters of lakes and rivers [3]. Animal foods are perishable but fish is ranked high among them and conventionally consumed as fresh in fishing areas due to complication in its handling, processing and perishable nature [4]. The quality deterioration of fish mostly occurs in fat and fish fat is mainly composed of unsaturated fatty acids. The environmental oxygen rapidly affects these unsaturated fatty acids which results in oxidation of these unsaturated fatty acids leading to deteriorate the quality of fish meat. The neutral pH, high amount of enzymes in the tissues, water holding capacity and connective tissues content also contribute to quality deterioration of fish [5].

Oxygen is responsible for degradation processes in foods like lipid oxidation, microorganism growth, enzymatic browning and vitamin loss [6]. The lipid oxidation result in development of off flavor, off color, nutrient loss [7] and oxidative processes causing degradation in meat proteins, pigment and lipids ultimately limiting the shelf life [8]. The highly perishable nature of fishery products demand the development of process for their better distribution in “as-is” condition. Edible coating is one of most promising fish preservation technique in this prospective with consumer interest potential [9]. Edible coating, a biodegradable material to be eaten as a part of food material without any side effects is the best choice for food industries to capture the consumer demands [10]. Edible coating is applied to the product surface and is responsible for restrict oxygen permeability, solute movement and serve as provider for a good barrier to moisture [11]. Whey protein fractions and isolates have been studied for film formation [12] and their potential as edible film forming agent during last decade due to favorable functional properties and dairy industry surplus [13]. The whey proteins have been used to produce transparent, flexible, colorless and odorless films [14] with moderate potential as moisture barrier and excellent oxygen barrier potential [15]. The making of protein-based films generally needs the incorporation of a minimal content of plasticizer to reduce its brittleness by weakening intermolecular forces between adjacent polymer chains. Plasticizers are essential for hydrocolloid coatings to enhance edible film processability and flexibility by raising the volume or molecular mobility of polymers and enhancing intermolecular spacing, by decreasing internal hydrogen bonding between polymer chains. Common plasticizers include polyols like sorbitol, glycerol, mannitol, propylene glycol and polyethylene glycol. The current study was designed with objective to apply whey protein based coating on fish fillets and assess its impact on oxidative stability and quality characteristics.

## Materials and methods

### Materials preparation

Fresh Rohu (*Labeo rohita*) was procured from University of Agriculture, Faisalabad, pakistan fisheries and all chemicals and reagents required to carry out research were purchased from Sigma Aldrich (Germany) and Merck (Germany). Fishes were cleaned to remove blood residues and dirt particles attached on the surface of fish and were cut into fillets of uniform sizes and stored at −18 °C prior to coating. Whey proteins concentrate based coating solutions were formulated by following the procedure as described by Diaz et al. (2011). Three coatings of different compositions were prepared: Whey + glycerol (1:2); Whey + sorbitol (1:2); Whey + glycerol + sorbitol (1:1:1). Protein coating solutions were prepared by slow stirring of whey protein concentrate (8 % protein; w/ w) in distilled water for 30 min at room temperature and later plasticizers were added as per treatment plan. Uniform homogenous consistency of coating solution was achieved by further stirring for 30 min and then heated in water bath at 80 °C for 30 min.

### Whey protein coating and storage of fish fillets

The edible coatings of whey proteins concentrate were applied to fish fillet dipping coating solutions for two minutes. After application of coating solution, these fish fillets were dried at room temperature for 15 min to maintain uniform thickness (1.5 mm) of coatings. Non-coated and whey protein concentrate coated samples were stored at −18 °C and were subjected of analysis at regular intervals (0, 10, 20, 30 and 40 days of storage).

### Coating retention

The yield after coatings were measured following the procedures described by Diaz et al. [16] The fish fillets were weighed before and after coating. The yield (weight gain, %) of coated fillets was calculated as:

$$ \mathrm{Coating}\ \mathrm{retention}\ \left(\%\right)=\frac{\mathrm{Wt}.\ \mathrm{o}\mathrm{f}\ \mathrm{coated}\ \mathrm{f}\mathrm{illet}\ \mathrm{pieces}}{\mathrm{Wt}.\ \mathrm{o}\mathrm{f}\ \mathrm{n}\mathrm{o}\mathrm{n}\hbox{-} \mathrm{coated}\ \mathrm{raw}\ \mathrm{f}\mathrm{illet}\ \mathrm{pieces}}\;\mathrm{x}\ 100 $$

### Thaw yield measurement

Thaw yield was measured according to method as described by Diaz et al. [16], frozen fillet were removed from the freezer, kept for 9 h at refrigerated temperature (5 °C), removed from the freezer bag, and placed on a rack for 2 min to release liquid drip. Then the thawed fillet pieces were weighed. The thaw yield (%) was determined by using the formula.

$$ \mathrm{Thaw}\ \mathrm{Yield}\ \left(\%\right)=\frac{\mathrm{Wt}.\ \mathrm{o}\mathrm{f}\ \mathrm{the}\ \mathrm{thawed}\ \mathrm{coated}\ \mathrm{f}\mathrm{illet}\ \mathrm{pieces}}{\mathrm{Wt}.\ \mathrm{o}\mathrm{f}\ \mathrm{the}\ \mathrm{n}\mathrm{o}\mathrm{n}\hbox{-} \mathrm{coated}\ \mathrm{raw}\ \mathrm{f}\mathrm{illet}\ \mathrm{pieces}} $$

### Drip loss measurement

Drip loss was obtained by following the method as descried by Diaz et al. [16]. After thawing, fish samples were suspended in a polyethylene bag sealed under atmospheric pressure. The sample was held at 2 °C for 24 h and then reweighed. The drip loss (%) was determined by using the formula.

$$ \mathrm{Drip}\ \mathrm{loss}\;\left(\%\right)=\frac{\left[\left(\mathrm{W}\mathrm{t}.\ \mathrm{of}\ \mathrm{t}\mathrm{he}\ \mathrm{frozen}\ \mathrm{fillet}\mathrm{s}\;\hbox{-}\;\mathrm{W}\mathrm{t}.\;\mathrm{of}\ \mathrm{t}\mathrm{he}\ \mathrm{Thawad}\;\mathrm{t}\mathrm{hawad}\right)\times 100\Big)\right]}{\mathrm{Wt}.\ \mathrm{of}\ \mathrm{t}\mathrm{he}\ \mathrm{frozen}\ \mathrm{coated}\ \mathrm{fillet}\ \mathrm{pieces}} $$

### Oil uptake measurement

Oil uptake of fish fillets was determined by the method as described by Kilincceker et al. [13] 30 g fish sample was weighed and deep fried in the cooking oil (Canola oil). Then fat content present in fried fish fillet was determined by using Soxhlet extractor. One gram oven dried sample was used to extract the fat content with n-Hexane. The n-Hexane was evaporated and recovered with the help of rotary evaporator. The fat content was weighed and oil uptake was calculated by the given formula.

$$ \mathrm{Oil}\ \mathrm{uptake}\ \left(\%\right) = \frac{\mathrm{Wt}.\ \mathrm{of}\ \mathrm{fried}\ \mathrm{sample}\mathit{\hbox{-}}\;\mathrm{W}\mathrm{t}.\ \mathrm{of}\ \mathrm{defatted}\ \mathrm{sample}}{\mathrm{Wt}.\;\mathrm{of}\;\mathrm{sample}} \times 100 $$

### pH measurement

The pH of fish fillets was estimated by homogenizing 10 g of sample in 100 mL distilled water prior and after coating fish fillets. Purposely, the samples were homogenized in 100 mL of distilled water for about 30s and pH was noted by placing the pH electrode in samples [17].

### Color analysis

Color values (L*, a*, and b*) of fish fillet samples were obtained by a colorimeter following the method as described by Diaz et al. [16] Observations were made for edible coated and non-coated samples at three different locations.

### Oxidative stability of fish fillets

The oxidative stability of the whey proteins concentrate coated fish fillets was determined by Peroxide Value and TBARS Value. Peroxide value of fish fillets was determined by adopting the procedure described by Shon & Chin [18]. 5 g sample was heated in a water bath for 3 min at 60 °C followed by thorough mixing through agitation in order to dissolve the fat and homogenize the sample after addition of 30 mL of acetic acid-chloroform solution (3:2 v/v). After filtration saturated potassium iodide solution was added in the filtrate @ 0.5 mL before transferring into a burette. The sample was titrated against a standard solution of sodium thiosulfate (25 g/L) using starch solution as indicator. The peroxide value was calculated and expressed in milliequivalent peroxides per kg of the fish sample:

$$ \mathrm{P}\mathrm{O}\mathrm{V}\;\left(\mathrm{m}\mathrm{e}\mathrm{q}/\mathrm{kg}\right)=\frac{S\times N}{W}\times 1000 $$

S = Volume of titration in mL

N = Normality of the sodium thiosulfate solution

W = Sample weight in kg

The TBARS value of the whey proteins concentrate coated fish fillets was assessed by measuring mg of malondialdehyde per kg of fish fillets by following the method as described by Luo et al. [19] Briefly 5 g of sample was weighed into 50-mL test tube and homogenized with 50 μL of butylated hydroxytoluene (7.2 %) and 15 mL of deionized distilled water using a homogenizer for 15 s at high speed. One milliliter of the meat homogenate was transferred to a disposable test tube (13 × 100 mm) and 2 mL added TBA/trichloroacetic acid solution (TCA; 15 mM TBA/15 % TCA). The mixture was vortex and incubated in a boiling water bath for 15 min to develop color. Then samples were cooled in the ice water for 10 min, vortexed again, and centrifuged for 15 min at 2,000 × g at 4 °C. The absorbance of the resulting supernatant solution was determined at 531 nm against a blank containing 1 mL of deionized distilled water and 2 mL of TBA/TCA solution. The amounts of TBARS were expressed as milligrams of malondialdehyde (MDA) per kilogram of fish fillets.

### Total volatile basic nitrogen (TVB-N) measurement

The total volatile basic nitrogen value of fish fillets was determined by the micro titration method as described by Sallama et al. [20] 10 g fish flesh sample was dispersed in 100 mL of distilled water by stirring for 30 min and filtered. Afterwards, 5 mL MgO (1 %) solution was added to 5 ml filtrate and Kjeldahl apparatus was used for sample distillation. The nitrogen released was absorbed in 20 mL aqueous solution of boric acid (2 %) containing methylene blue indicator. The trapped nitrogen was titrated with 0.01 M HCl solution and TVB-N content of samples was measured through equation using the volume of 0.01 M HCl used for titration.

### Sensory evaluation of fish fillets

The sensory evaluation of fried fish fillets was conducted for color, odor, taste, texture and overall acceptability by a panel of judges using 9-point hedonic scale according to the procedure of Meilgaard et al [21].

### Statistical analysis

The data obtained for each parameter was subjected to statistical analysis to determine the level of significance according to the method described by Steel et al. [22] by using the software package (Statistic 8.1). The Duncan’s multiple range (DMR) test was used to estimate the level of significance existed between mean values.

## Results and discussion

### Quality assessment of whey protein coated rohu fillets

The coating retention of fish fillets was assessed by measuring the weight gain of fish fillets after application of whey proteins concentrate coating (Table 1). It is evident from results that weight gain did not differ significantly for different whey proteins concentrate coating indicating the uniformity of coating application for all coating types. These findings are in agreement with the previous work of Diaz et al. [16] who observed non-significant difference in weight gain of whey protein based edible coated Atlantic salmon (*Salmo salar*). Thaw yield is measure of fish weight achieved after thawing of fish fillet and yield was recorded higher for the treatments with coating as compared to control as depicted in Table 2. The un-coated samples (T_0_) results in higher thawing losses as lowest thaw yield was observed for those samples. Thaw yield of frozen fish fillet also decreased with storage time and all samples showed similar decreasing trend in thaw yield with storage. However, coating samples have higher yield than un-coated fish fillets indicating that coatings have moisture barrier property and thus preserve the weight of the fish fillets. Our findings are in harmony with the previous work of Valverde et al. [23] who stated that aloevera based novel coatings served as a good preservative agent to maintain the quality and safety of poultry meat during cold storage. Diaz et al. [16] reported similar trend in Atlantic salmon (*salmo salar*) fish during frozen storage. The correlated factor with thaw yield is drip loss, as the product having higher thaw yield showed have minimum drip loss. The results explicated in Table 2 indicate that T_3_ have minimum mean drip loss (0.69 %) as compare to other coated and non-coated samples (1.28 %). Drip loss occurred due to moisture removal from frozen fish fillets with the passage of time. Whey protein coating having glycerol and sorbitol in 1:1 present highest barrier against moisture loss and thus reducing drip loss of coated fish fillets. These findings are in accordance with results of Han et al. [24] who applied chitosan coating and observed reduced drip loss. Edible coating reduced the amount of oil uptake during the frying process as oil use same channel of water removal from sample for uptake during frying. Thus, reduced water removal as a result of coating reduces the oil uptake by coated samples. The samples having lower drip losses have lower oil uptake as indicated in Table 3. These result are in agreement with findings of Elmadfa et al. [25] who observed similar trend in edible coated farmed fish (*salmo salar)* during frying process*.*

**Table 1:** Coating retention (%) of whey protein edible coating by Rohu fish fillets

Treatments	Yield
T_1_	102.48 ± 2.05
T_2_	102.39 ± 2.05
T_3_	102.59 ± 2.05

T_1_: Whey: Glycerol (1:2) T2: Whey: Sorbitol (1:2) T3: Whey: Glycerol: Sorbitol (1:1:1)

**Table 2:** Thaw yield and Drip loss of whey protein coated rohu fish fillets

Days	Thaw yield (%)				Drip loss (%)			
	T_0_	T_1_	T_2_	T_3_	T_0_	T_1_	T_2_	T_3_
10	98.59 ± 1.97	101.53 ± 2.03	101.52 ± 2.03	101.92 ± 2.04	1.301 ± 0.03	0.927 ± 0.02	0.849 ± 0.02	0.653 ± 0.01
20	97.35 ± 1.95	100.57 ± 2.01	100.63 ± 2.01	101.23 ± 2.02	1.25 ± 0.02	0.945 ± 0.02	0.876 ± 0.02	0.677 ± 0.01
30	96.11 ± 1.92	99.59 ± 1.99	99.73 ± 1.99	100.51 ± 2.01	1.27 ± 0.03	0.974 ± 0.02	0.894 ± 0.02	0.711 ± 0.01
40	94.84 ± 1.90	98.60 ± 1.97	98.81 ± 1.98	99.77 ± 2.00	1.31 ± 0.03	0.994 ± 0.02	0.922 ± 0.02	0.736 ± 0.01
Mean	96.72 ± 1.93b	100.07 ± 2.00a	100.17 ± 2.00a	100.86 ± 2.02a	1.283 ± 0.03a	0.960 ± 0.02b	0.885 ± 0.02c	0.694 ± 0.01d

The values are mean ± SD of three independent measurements, Means sharing similar letter in a row are statistically non-significant (*P* > 0.05)

T_0_: Without coating T_1_: Whey: Glycerol (1:2) T2: Whey: Sorbitol (1:2) T3: Whey: Glycerol: Sorbitol (1:1:1)

**Table 3:** Oil uptake and pH of whey protein coated rohu fish fillets

Days	Oil uptake (%)				pH			
	T_0_	T_1_	T_2_	T_3_	T_0_	T_1_	T_2_	T_3_
10	18.00 ± 0.36	15.12 ± 0.30	15.01 ± 0.30	13.72 ± 0.27	6.18 ± 0.12	6.16 ± 0.12	6.16 ± 0.12	6.17 ± 0.12
20	18.10 ± 0.36	15.22 ± 0.30	15.11 ± 0.30	13.82 ± 0.28	5.73 ± 0.11	5.77 ± 0.12	5.81 ± 0.12	5.86 ± 0.12
30	18.19 ± 0.36	15.37 ± 0.31	15.26 ± 0.31	13.94 ± 0.28	6.38 ± 0.13	6.15 ± 0.12	6.14 ± 0.12	6.11 ± 0.12
40	18.25 ± 0.36	15.57 ± 0.31	15.44 ± 0.31	14.08 ± 0.28	7.06 ± 0.14	6.55 ± 0.13	6.49 ± 0.13	6.37 ± 0.13
Mean	18.17 ± 0.36a	15.42 ± 0.31b	15.29 ± 0.31b	13.95 ± 0.28c	6.63 ± 0.13a	6.32 ± 0.13b	6.29 ± 0.13b	6.23 ± 0.12c

The values are mean ± SD of three independent measurements, Means sharing similar letter in a row are statistically non-significant (*P* > 0.05)

T_0_: Without coating T_1_: Whey: Glycerol (1:2) T2: Whey: Sorbitol (1:2) T3: Whey: Glycerol: Sorbitol (1:1:1)

### pH and color of whey protein edible coated rohu fillets

The pH of food is consider an important attribute affecting its quality and shelf stability by regulating many functions and reactions. The data explicated in Table 3 showed significant variation in pH of fish fillet as a result of edible whey protein coating. The results indicated an ultimate decrease in pH of coated fish fillets with whey protein coating. The pH of sample showed an initial decrease and then increase in pH value. This increase in pH was attributed to production of volatile bases by the degradation of proteins and initial decrease of pH was linked to dissolution of CO_2_. Amongst the treatments, the maximum pH increase was found in T_0_ (uncoated) and the lowest pH increase was documented in T_3_ (Whey (1): glycerol (1): sorbitol (1) ). The samples exhibited a gradual increase in the pH ranging from 6.16 ± 0.12 to 7.05 ± 0.14 during 40 days of frozen storage and sample exhibited a similar trend that maximum increased was observed in T_0_ during storage while lowest was found for T_3_. The results are supported by earlier findings of Fan et al. [26] who reported an increase in pH of silver carp fish fillets associated with production of volatile bases due to the degradation of protein to ammonia and trimethylamine by microbial or endogenous enzymes. Vargas et al. [9] reported an increase in pH with the advancement of storage period of fish fillets.

The appearance of foods is one of the major determinants of its appeal to consumers and consequently the sales of product. Oxidative processes in meat lead to the degradation of lipids and proteins which, in turn, contribute to the deterioration in flavor, texture and color of displayed meat [27]. The results regarding mean values for color L*, a* and b* values of coated fish fillets are given in Table 4. It is evident from the results that whey protein coating significantly affect the color L*, a* and b* values of fish fillets. The storage period of edible coated fish fillets also significantly increase the L*, a* and b* value fish fillets. It is evident from results that lower degradation of color values of fish fillets was observed in whey protein coated samples and this degradation was lowest in whey protein coating prepared by combination of plasticizers. These results are in compliance with the findings reported by Diaz et al. [16] who reported similar trend in Atlantic salmon (*salmo salar*) fish during frozen storage. The different factors are responsible for color change in fish meat.

**Table 4:** Color L*, a* and b* values of whey protein coated rohu fish fillets

Days		T_0_	T_1_	T_2_	T_3_	Mean
0	L	41.38 ± 0.83^p^	43.98 ± 0.88^lm^	43.14 ± 0.86^mno^	42.09 ± 0.84^nop^	42.30 ± 0.85^e^
	a	7.83 ± 0.16l^m^	7.49 ± 0.15^n^	7.50 ± 0.15^mn^	7.29 ± 0.15^n^	7.55 ± 0.15^e^
	b	5.96 ± 0.12^p^	5.79 ± 0.12^p^	5.82 ± 0.12^p^	5.73 ± 0.11^p^	5.85 ± 0.12^e^
10	L	44.08 ± 0.88^lm^	45.38 ± 0.91^i-l^	44.5 ± 0.89^klm^	43.28 ± 0.87^mno^	43.98 ± 0.88^d^
	a	9.73 ± 0.19^i^	8.59 ± 0.17^k^	8.55 ± 0.17^k^	8.09 ± 0.16^l^	8.74 ± 0.17^d^
	b	7.76 ± 0.16^m^	6.99 ± 0.14^n^	6.92 ± 0.14^n^	6.63 ± 0.13°	7.06 ± 0.14^d^
20	L	46.89 ± 0.94^fgh^	46.81 ± 0.94^f-i^	45.87 ± 0.92^g-k^	44.46 ± 0.89^klm^	45.69 ± 0.92^c^
	a	11.68 ± 0.23^e^	9.74 ± 0.19^i^	9.64 ± 0.19^i^	8.93 ± 0.18^j^	9.97 ± 0.20^c^
	b	9.61 ± 0.19^fg^	8.21 ± 0.16^kl^	8.04 ± 0.16^l^	7.55 ± 0.15^m^	8.31 ± 0.17^c^
30	L	49.81 ± 1.00^ab^	48.27 ± 0.97^cdef^	47.27 ± 0.95^efg^	45.69 ± 0.91^h-k^	47.46 ± 0.95^b^
	a	13.67 ± 0.27^b^	10.93 ± 0.22^fgh^	10.74 ± 0.21^gh^	9.81 ± 0.20^i^	11.23 ± 0.22^b^
	b	11.50 ± 0.23^b^	9.46 ± 0.19^g^	9.13 ± 0.18^i^	8.49 ± 0.17^j^	9.59 ± 0.19^b^
40	L	52.8 ± 1.06^a^	49.76 ± 1.00^bc^	48.68 ± 0.97^b-e^	46.94 ± 0.94^fgh^	49.26 ± 0.99^a^
	a	15.72 ± 0.31^a^	12.17 ± 0.24^cd^	11.86 ± 0.24^de^	10.70 ± 0.21^h^	12.53 ± 0.25^a^
	b	13.44 ± 0.27^a^	10.71 ± 0.21^d^	10.23 ± 0.20^e^	9.44 ± 0.19^gh^	10.88 ± 0.22^a^

The values are mean ± SD of three independent measurements, Means sharing similar letter in a row and column are statistically non-significant (*P* > 0.05)

T_0_: Without coating T_1_: Whey: Glycerol (1:2) T2: Whey: Sorbitol (1:2) T3: Whey: Glycerol: Sorbitol (1:1:1)

### Oxidative stability of whey protein edible coated rohu fillets

Autoxidation is a major process of quality deterioration in food containing fats that leads towards generation of free radical and ultimately it spoils the quality by generation of off flavors and bad taste [28]. Lipid oxidation is mainly responsible for quality degradation meat and meat products during storage and it can be delayed or inhibited by the edible coating of food [29].

Peroxide value (POV) is an important indicator to measure rancidity of fat. The oxidation of fat produced free radicals that leads to the formation of aldehydes and ketones that decreases the quality of meat. It is evident from results (Table 5) that coatings and storage significantly affected peroxide value among treatments. The mean values for peroxide value of fish fillets showed that the highest oxidation occurred in non-coated samples (T_0_) while minimum fat degradation (2.42 ± 0.05 meq/kg) in samples coated with whey protein coating prepared with combination of plasticizers. The mean values for peroxide value of fish fillets changed from 0.92 ± 0.02 meq/kg at 0 day to 5.12 ± 0.10 meq/kg on 40^th^ day of storage. These findings showed that fish fillets treated with coatings especially T_3_ proved to be effective in retarding the lipid oxidation. Lipid oxidation occurred due to enzymatic activity in fish meat which can be triggered by availability of free oxygen. Thus, coating mixture provide barrier against oxygen penetration resulting in delayed lipid oxidation. These results are in compliance with the findings of Sallam [30] who also indicated a decline in peroxide value in salmon fish fillets under frozen storage.

**Table 5:** Peroxide Value (meq/kg) and TBARS Value (mg MDA/kg) of whey protein coated rohu fish fillets

Days	POV (meq/kg)					TBARS Value (mg MDA/kg)				
	T_0_	T_1_	T_2_	T_3_	Mean	T_0_	T_1_	T_2_	T_3_	Mean
0	0.92 ± 0.02	0.93 ± 0.02	0.90 ± 0.02	0.91 ± 0.02	0.92 ± 0.02e	0.18 ± 0.14	0.17 ± 0.06	0.18 ± 0.00	0.170 ± 0.00	0.175 ± 0.01e
10	2.42 ± 0.05	1.88 ± 0.04	1.76 ± 0.04	1.66 ± 0.03	1.93 ± 0.06d	0.48 ± 0.01	0.30 ± 0.01	0.28 ± 0.01	0.250 ± 0.01	0.327 ± 0.02d
20	3.97 ± 0.08	2.84 ± 0.06	2.64 ± 0.05	2.38 ± 0.05	2.96 ± 0.05c	0.83 ± 0.02	0.44 ± 0.01	0.40 ± 0.01	0.340 ± 0.01	0.502 ± 0.02c
30	5.57 ± 0.11	3.83 ± 0.08	3.53 ± 0.07	3.18 ± 0.06	4.03 ± 0.07b	1.13 ± 0.02	0.60 ± 0.01	0.540 ± 0.01	0.440 ± 0.01	0.677 ± 0.03b
40	7.19 ± 0.14	4.86 ± 0.10	4.44 ± 0.09	3.98 ± 0.08	5.12 ± 0.1a	1.48 ± 0.03	0.78 ± 0.02	0.690 ± 0.01	0.560 ± 0.01	0.877 ± 0.03a
Mean	4.01 ± 0.4a	2.868 ± 0.06b	2.65 ± 0.05b	2.42 ± 0.05c						

The values are mean ± SD of three independent measurements, Means sharing similar letter in a row and column are statistically non-significant (*P* > 0.05)

T_0_: Without coating T_1_: Whey: Glycerol (1:2) T2: Whey: Sorbitol (1:2) T3: Whey: Glycerol: Sorbitol (1:1:1)

The thiobarbituric acid reactive substance (TBARS) is used as an index of lipid oxidation measurement in meat and allied products. The results regarding TBARS of coated fish fillets in (Table 5) showed momentous effect coating on oxidation of fish fillets. The lowest oxidation of fat occurred in T_3_ and highest oxidation occurred in T_0_ (control). The lowest mean value for thiobarbituric acid 0.352 mg of MDA/kg was observed for T_3_ while control samples has higher TBARS mean value (0.820 ± 0.02 mg of MDA/kg). The TBARS value of fish fillets showed a gradual increase as 0.178 ± 0.00 mg of MDA/kg (0d) increased significantly to 0.867 ± 0.02 mg of MDA/kg after 40 days of storage. The results concluded that among the fish fillets treated with various composition of coating, whey protein coating prepared by combination of plasticizer (T_3_) has proved to be effective in retarding the lipid oxidation. The protein based coatings consisting oxidation by preventing or retarding the interaction between air and meat surface resulting in lower TBARS values. These results are in lined with findings of Diaz et al. [16] who reported that TBARS values of Atlantic salmon (*salmosalar*) fish coated with whey protein decreased as compared to control during frozen storage. Villegas et al. [31] reported lower TBARS value for coated bacons compared to control. Kilincceke et al. [13] also indicated lower TBARS value of coated fish fillet sample than that of the un-coated ones.

### Total volatile basic nitrogen of whey protein edible coated rohu fillets

Total volatile basic nitrogen is widely used as an indicator of meat spoilage that is mainly composed of ammonia, primary, secondary and tertiary amines produced by the degradation of protein and non-protein nitrogenous compounds. Protein degradation can occur in meat and meat based products due to microbial activity during storage. The 35-40 mg/100 g of TVB-N level in fish fillets is an indicative of spoiled fish. The results (Table 6) revealed a momentous effect of coatings on total volatile basic nitrogen of fish fillets. The storage of fish fillets also significantly affected total volatile basic nitrogen. The means revealed that maximum total volatile basic nitrogen (12.60 ± 0.25 mg/100 g) was observed in T_0_ (control) while lowest TVB-N (8.81 ± 0.18 mg/100 g) was exhibited by T_3_. A gradual increase in TVB-N ranging from 6.70 ± 0.13 to 13.43 ± 0.26 mg/100 g from 0 to 40^th^ day was observed during storage for 40 days at frozen state. Among treatments, the maximum increase was observed in T_0_ while the lowest TVB-N increase was found in T_3._ These results are in concordant with Mchugh and Senesi [11] who reported a gradual increase in TVB-N mainly due to production of amines during storage.

**Table 6:** Total volatile basic nitrogen (TVB-N) of whey protein coated rohu fish fillets

Days	T_0_	T_1_	T_2_	T_3_	Mean
0	6.7 ± 0.13	6.70 ± 0.13	6.7 ± 0.13	6.7 ± 0.13	6.70 ± 0.13e
10	9.6 ± 0.19	8.10 ± 0.16	8.0 ± 0.16	7.8 ± 0.16	8.38 ± 0.18d
20	12.6 ± 0.25	9.5 ± 0.19	9.3 ± 0.19	8.8 ± 0.18	10.05 ± 0.31c
30	15.6 ± 0.31	10.9 ± 0.22	10.6 ± 0.21	9.9 ± 0.20	11.75 ± 0.41b
40	18.6 ± 0.37	12.3 ± 0.25	11.9 ± 0.24	10.9 ± 0.22	13.43 ± 0.26a
Mean	12.6 ± 0.25a	9.5 ± 0.14b	9.3 ± 0.14b	8.81 ± 0.18c	

T_0_: Without coating T_1_: Whey: Glycerol (1:2) T2: Whey: Sorbitol (1:2) T3: Whey: Glycerol: Sorbitol (1:1:1)

The values are mean ± SD of three independent measurements, Means sharing similar letter in a row and column are statistically non-significant ( P > 0.05)

### Sensory evaluation of whey protein edible coated rohu fillets

Sensory evaluation of food can be defined as “a scientific method used to evoke, measure, analyze and interpret responses to products as perceived through the senses of sight, touch, smell, taste, and hearing”. It is an important tool to access the quality and consumer acceptability of a food product. The sensory parameters have significant effect on the rohu fish fillet and the coated samples have better sensory characteristics as compared to un-coated samples. Color is the first perception of eye perceived by the consumer and is main criteria for consumer to evaluate a product. The results (Table 7) showed whey based protein coatings with plasticizers significantly improved the color of fish fillets and color values decreased with the advancement of storage period as perceived by panel of judges. The results of the present study are in consistent with the findings of Paulo et al. [32] who also reported that color values of fish are decreased with the advancement of storage time. Odor is an important parameter in sensory evaluation of a food product. The results (Table 7) showed that whey based protein coatings with plasticizers significantly affected odor of fish fillets and odor values were decreased with the advancement of storage period. The findings are in harmony with the findings of Del-Valle et al. [33] who used cactus mucilage based coatings and applied on fish fillets and reported a decrease in odor scores with the advancement of storage period. Taste resides at the core of desirability of any food product. The results (Table 6) showed that whey based protein coatings with plasticizers significantly affected the taste of fish fillets and taste values were decreased with the advancement of storage period. These results regarding change in taste are in line with the findings of Vaithiyanathan et al. [34] who reported a decrease in taste of edible coated meat with the advancement of storage periods due to the enzymatic degradation. Texture is a very important attribute in the sensory evaluation of meat products and is an important indicator of consumer perception regarding the quality and acceptability of meat. The results (Table 7) showed that whey based protein coatings significantly affected the texture of fish fillets and texture values decreased with the advancement of storage period. The results of present study are consistent with the findings of Devatkal et al. [35] and Lu et al. [36] who reported an increase in hardness of fish meat with the progression of storage period. The results (Table 7) showed that whey based protein coatings with different plasticizers significantly affect the overall acceptability of the fish fillets and overall acceptability scores decreased with the advancement of storage period. The results of current investigation are in accordance with that of Hambleton et al. [37] and Song et al. [38] who reported that overall acceptability of fish fillets coated with alginate coatings were improved as compared to control.

**Table 7:** Sensory attributes of whey protein coated rohu fish fillets

	Days	T_0_	T_1_	T_2_	T_3_
Color	0	8.70 ± 0.17^a-c^	8.72 ± 0.17^ab^	8.69 ± 0.17^a-c^	8.78 ± 0.18^a^
	10	7.90 ± 0.16^ij^	8.47 ± .017^b-e^	8.46 ± 0.17^c-e^	8.58 ± 0.17^a-d^
	20	7.05 ± 0.14^no^	8.17 ± 0.16^f-h^	8.20 ± 0.16^fg^	8.34 ± 0.17^d-f^
	30	6.15 ± 0.12^q^	7.82 ± 0.16^ij^	7.90 ± 0.16^ij^	8.06 ± 0.16^g-i^
	40	5.20 ± 0.10^r^	7.42 ± 0.15^lm^	7.56 ± 0.15^kl^	7.73 ± 0.15^jk^
Odor	0	8.80 ± 0.18^a-c^	8.83 ± 0.18^a^	8.79 ± 0.18^a-c^	8.88 ± 0.18^a^
	10	8.00 ± 0.16^gh^	8.56 ± 0.17^b-e^	8.54 ± 0.17^c-e^	8.70 ± 0.17^a-d^
	20	7.15 ± 0.14^k^	8.25 ± 0.16^fg^	8.27 ± 0.17^f^	8.50 ± 0.17^d-f^
	30	6.25 ± 0.13^m^	7.90 ± 0.16^hi^	7.98 ± 0.16^h^	8.27 ± 0.17^f^
	40	5.30 ± 0.11^n^	7.41 ± 0.15^jk^	7.66 ± 0.15^ij^	7.99 ± 0.16^gh^
Taste	0	8.65 ± 0.17^a-c^	8.69 ± 0.17^ab^	8.67 ± 0.17^a-c^	8.70 ± 0.17^a^
	10	7.83 ± 0.16^i^	8.42 ± 0.17^c-e^	8.44 ± 0.17^b-e^	8.55 ± 0.17^a-d^
	20	6.96 ± 0.14^l^	8.12 ± 0.16^f-h^	8.19 ± 0.16^ef^	8.37 ± 0.17^d-f^
	30	6.03 ± 0.12^m^	7.79 ± 0.16^ij^	7.89 ± 0.16^hi^	8.16 ± 0.16^fg^
	40	5.05 ± 0.10^n^	7.41 ± 0.15^k^	7.56 ± 0.15^jk^	7.91 ± 0.16^g-i^
Texture	0	8.75 ± 0.18^ab^	8.78 ± 0.18^ab^	8.76 ± 0.18^ab^	8.79 ± 0.18^a^
	10	7.95 ± 0.16^gh^	8.55 ± 0.17^abcd^	8.52 ± 0.17^bcde^	8.60 ± 0.17^abc^
	20	7.10 ± 0.14^jk^	8.28 ± 0.17^ef^	8.25 ± 0.16^f^	8.39 ± 0.17^cdef^
	30	6.20 ± 0.12^m^	7.99 ± 0.16^gh^	7.96 ± 0.16^gh^	8.15 ± 0.16^fg^
	40	5.25 ± 0.11^n^	7.66 ± 0.15^i^	7.63 ± 0.15^i^	7.89 ± 0.16^ghi^
Acceptability	0	8.70 ± 0.17^ab^	8.71 ± 0.17^a^	8.70 ± 0.17^ab^	8.75 ± 0.18^a^
	10	7.92 ± 0.16^gh^	8.50 ± 0.17^a-d^	8.45 ± 0.17^b-e^	8.60 ± 0.17^a-c^
	20	7.05 ± 0.14^kl^	8.20 ± 0.16ef	8.22 ± 0.16^ef^	8.40 ± 0.17^c-f^
	30	6.05 ± 0.12^n^	7.85 ± 0.16^h^	7.90 ± 0.16^gh^	8.15 ± 0.16^fg^
	40	5.20 ± 0.10°	7.45 ± 0.15^j^	7.55 ± 0.15^ij^	7.85 ± 0.16^h^

The values are mean ± SD of three independent measurements, Means sharing similar letter in a column are statistically non-significant (*P* > 0.05)

T_0_: Without coating T_1_: Whey: Glycerol (1:2) T2: Whey: Sorbitol (1:2) T3: Whey: Glycerol: Sorbitol (1:1:1)

## Conclusion

Whey protein concentrate coating play effective role in improving the quality attributes of rohu (*Labeo rohita*) fillets, protecting fish fillets from oxidative and microbial spoilage. Whey protein coating with combination of plasticizers (Whey: Glycerol: Sorbitol 1:1:1) showed better result in improvement of quality attributes and retarding oxidative degradation, However, certain quality improver or additives can be used in future studies to prevent changes in sensory quality of coated fish fillets.
